# Female Reproductive Decline Is Determined by Remaining Ovarian Reserve and Age

**DOI:** 10.1371/journal.pone.0108343

**Published:** 2014-10-13

**Authors:** Pawel Wilkosz, Gareth D. Greggains, Tom G. Tanbo, Peter Fedorcsak

**Affiliations:** 1 Section for Reproductive Medicine, Department of Gynecology, Oslo University Hospital Rikshospitalet, Oslo, Norway; 2 Institute of Clinical Medicine, University of Oslo, Oslo, Norway; University Hospital of Münster, Germany

## Abstract

The early decline and loss of female fertility in humans and other species represents an evolutionary paradox. Despite being born with a vast stock of oocytes, females encounter an exhaustion of ovarian reserve and sterility half way through their natural lives. Female reproductive ageing has been proposed to proceed as an ongoing decline in ovarian reserve, determined by remaining ovarian follicle number. However, despite extensive modelling, the respective contributions of intra-, inter-, and extra-ovarian signalling have not been fully characterised. It remains unclear whether reproductive ageing progresses simply as a pre-determined function of remaining ovarian follicles, or as an age-dependent process in humans. Here, we have analysed ovarian response to hormonal stimulation in women who have undergone surgical removal of a single ovary, in order to investigate the relative contributions of intra-, inter, and extra-ovarian signalling on reproductive ageing. Our data show that in unilaterally oophorectomised women, ovarian response to follicle stimulating hormone (FSH) declines beyond levels predicted by a total ovarian follicle pool model of reproductive ageing. Maintenance of ovarian function later in reproductive life, despite the removal of half of the total ovarian reserve, suggests a role for an extra-ovarian age-dependent regulation of reproductive decline. This highlights the need for further work to identify signalling factors that communicate age-related signals between the soma and the germline.

## Introduction

Women are born with a finite stock of approximately 500 000–1 000 000 follicle-enclosed oocytes, which are depleted over the course of their lives [1]–[5]. Only 1% of these oocytes are ever ovulated, with the vast majority being lost to follicle atresia [6]. Decline in the ovarian follicle pool, defined at any time as the remaining cohort of ovarian follicles, is associated with reduced fertility in the mid-thirties, irregular menstruation from the mid-forties, and finally, follicle exhaustion and menopause in the early fifties [7], [8]. This process represents one of the earliest organ failures in natural female ageing and raises intriguing questions regarding the relationship between reproductive and organismal ageing [9].

In humans, waves of follicles are recruited to growth throughout reproductive life, even during early pregnancy and use of contraceptive pills [10], [11]. Follicle recruitment varies substantially with age, from more than 1400 per month in the early twenties to fewer than 30 in the late forties [12]. A monthly selection process by FSH gives rise to a single dominant follicle, which is ovulated during the same cycle [6].

The huge oocyte attrition observed in humans is brought about by follicle atresia, which continues throughout life [6]. Atresia occurs before or after recruitment of follicles to growth, in a menstrual cycle-independent or dependent way. The ratio of these two stages of atresia shifts during life, so that relatively more non-growing follicles are lost in young women and fewer in older women [13], [14]. However, it is unknown how these changing follicle dynamics are regulated during ageing.

Conventional models characterise reproductive ageing as being dependent on the remaining ovarian reserve or follicle pool [2], [15]. However, whether this pool exists as two independent ovarian units or as a combined total ovarian follicle pool, regulated by inter-ovarian signalling, has not been fully considered. In addition, the relationship between reproductive and organismal ageing in humans has received little attention. Models include age parameters to fit data, but fall short of a causation, and the importance of inter-ovarian endocrine signalling is unclear [2], [4], [15], [16].

Direct longitudinal study of follicle reserve and growth is not possible in humans. However, the effect of an artificial reduction of ovarian follicle reserve can give insight into these processes. Partial bilateral removal of ovarian tissue is rarely conducted in clinical practice, and is difficult to interpret due to the challenges in quantifying the removed fraction of follicles, the effect of underlying ovarian disease, and damage to the ovary from the procedure. Some groups have investigated the effect of gonadotoxic treatment for cancer on fertility and menopause, but uncertainty regarding the proportional loss of ovarian follicles and damage sustained to remaining follicles from treatment makes interpretation of these data difficult.

Here, we have uncoupled intra- and inter-ovarian signalling effects on reproductive ageing by retrospectively examining ovarian response in patients who have undergone unilateral oophorectomy. We have used ovarian response to follicle stimulating hormone (FSH), during assisted reproductive treatment, to examine these effects. Response, measured as the number of oocytes retrieved after controlled ovarian stimulation, is dependent on the number of selectable follicles available, which in turn, is related to the available ovarian follicle reserve [17]. In addition, sectional and longitudinal analysis of ovarian response has allowed us to uncover long-term age-specific effects on follicle dynamics during ovarian decline.

## Materials and Methods

### Ethics Statement

Permission for use of clinical data for this study has been granted by the data protection officer at Oslo University Hospital Rikshospitalet, license no. 08/3438. Patients records were anonymized prior to analysis.

### Patients

Patients were selected from a cohort of 6844 women who had undergone IVF or ICSI treatment at Rikshospitalet between 1996 and 2012. Patients with PCOS and two patients with incomplete records were excluded. All treatments were preceded by controlled ovarian stimulation, as described previously [18], [19]. The study group consisted of 97 patients who had undergone unilateral oophorectomy prior to treatment start. Indications for oophorectomy are shown in Table S1 in File S1. The control group consisted of 6747 patients after exclusion of PCOS patients and those who had undergone any type of ovarian surgery, such as cystectomy, ovarian resection or ablation on one or both ovaries.

Patients that had undergone unilateral oophorectomy due to endometriosis were included, as the presence and/or extent of endometriosis in the remaining ovary was not known and mild endometriosis does not significantly impair fertility [18]. Whilst, it has been documented that endometriomas and peritoneal endometriosis might reduce ovarian reserve followed by decreased ovarian response during IVF treatment [20], [21], [22], we found no differences in median ovarian response during the first cycle and median age between women with or without endometriosis in the unilateral oophorectomized group (2.33 vs 2.78 and 33.3 vs 33.7 years, respectively).

Patients in the unilateral oophorectomy group underwent 201 cycles of IVF or ICSI, whereas patients in the control group received 13.800 treatments (approximately two treatment cycles per patient in each groups). Patient characteristics are outlined in Table S2 in File S1.

Blood samples for serum AMH assays were drawn randomly during the menstrual cycle, and were centrifuged within 45 minutes of collection and stored at −70°C until assay with the Active MIS/AMH ELISA kit (DSL-10-14400, Diagnostic Systems Laboratories Inc., TX, USA). The limit of detection was 0.006 ng/ml and the intra and inter-assay coefficients of variations were 5.2% and 9.1%, respectively.

### Construction of the model of prediction of reproductive age

**Figure 1:**
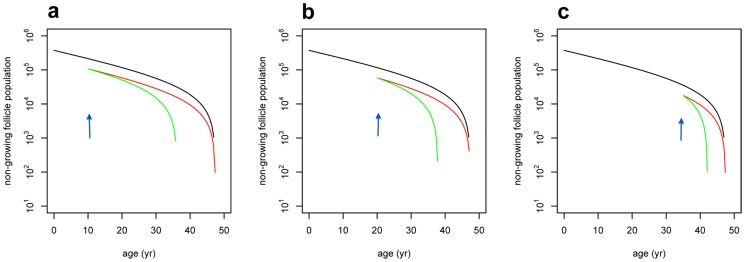
Modelling of decline in ovarian reserve. **a–c.** Calculated trajectories of follicle loss after unilateral oophorectomy at ages 10 years (a), 20 years (b), and 35 years (c), assuming the total ovarian follicle pool (green) or alternative model of decline of ovarian reserve (red). Arrows on figures c,d and e indicate time of unilateral oophorectomy at the respective ages.

Age-dependent number of human NGFs were calculated based on Kelsey and Wallace's quantitative histological data of a non-growing follicle population [4] covering samples from 288 women between 0–51 years of age (Figure 1). The decay model of Faddy [14] postulates that the rate of natural NGF depletion is proportional to the number of remaining follicles in the ovary, more specifically it can be described with the differential equation:
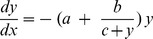
where *a*, *b*, *c* are constants, *x* denotes age and *y* the number of NGF. The equation was solved for *y* and fitted on the Wallace and Kelsey data-set using non-linear regression. Using this, we generated curves for reproductive decline based on age at oophorectomy and two models of reproductive ageing (Figure 1).

Predicted reproductive age after oophorectomy was calculated by assuming 50% sudden reduction of the non-growing follicle (NGF) population at the age of oophorectomy, and subsequent individual follicle loss was predicted by the power model, based on data from Hansen et al. [2] (Table 1). Using this model, we predicted the advancement of reproductive age at which a sudden halving of follicle number would equal the natural decline of NGF in that woman (Table 1).

**Table:** **Table 1.** Ovarian response during gonadotropin stimulation for IVF among women with two intact ovaries and women who had undergone unilateral oophorectomy.

Age (years)	Two intact ovaries			Unilateral oophorectomy						
	n	Age	No. of collected oocytes per 1000 IU total FSH dose	n	Age	No. of collected oocytes per 1000 IU total FSH dose	Age at oophorectomy	Predicted* current reproductive age	Expected No. of collected oocytes per 1000 IU total FSH dose at predicted* reproductive age	P-value†
21–32	2415	29.2 (SD 2.1)	5.10	38	29.3 (SD 2.0)	2.57	24.2 (SD 4.9)	34.9 (SD 2.0)	3.77	0.02
32–35	1878	33.5 (SD 0.9)	4.23	29	33.8 (SD 0.9)	2.04	27.8 (SD 5.1)	38.5 (SD 1.5)	2.81	0.15
35–46	2247	37.3 (SD 1.6)	3.3	30	37.2 (SD 1.8)	2.17	30.2 (SD 7.8)	41.9 (SD 3.0)	2.13	0.02
Total	6540	33.2 (SD 3.7)	4.09	97	33.1 (SD 3.7)	2.27	27.1 (SD 6.5)	38.1 (SD 3.6)	2.90	0.03

Data are geometric mean or mean, SD. *, assuming the power model of Hansen [2]. †, paired-sample t-test comparing observed and expected oocyte count per 1000 IU total FSH dose.

### Statistical analysis

**Figure 2:**
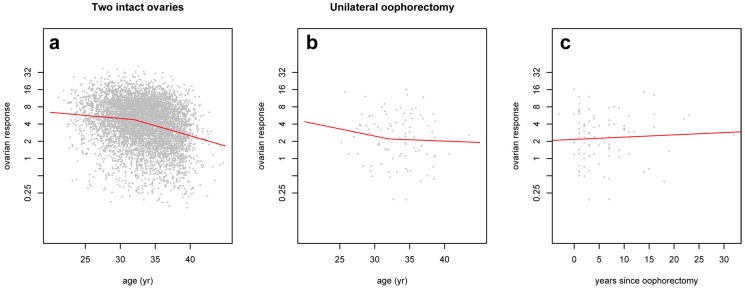
Ovarian response after unilateral oophorectomy. Two-piece exponential decay model of ovarian response in women with intact ovaries *(* ***a*** *)*, in women who had undergone unilateral oophorectomy according to age *(* ***b*** *)*, and time since oophorectomy *(* ***c*** *)*. Ovarian response is expressed as ratio of number of collected oocytes and 1000 IU total FSH dose during ovarian stimulation for IVF.

**Figure 3:**
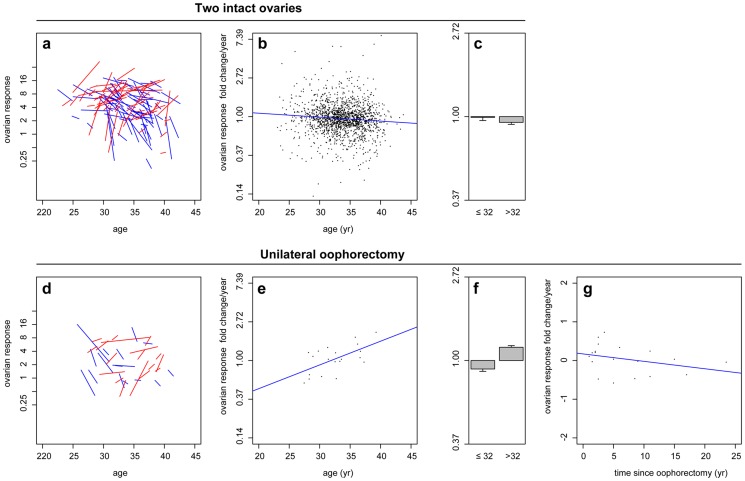
Longitudinal ovarian response after unilateral oophorectomy. Individual changes in ovarian response with age in women with two intact ovaries (**a**) and after unilateral oophorectomy (**d**). Ovarian response change/year in women with intact ovaries (**b, c**), and after unilateral oophorectomy (**e, f**). Ovarian response change/year in unilaterally oophorectomised women according to time after operation (**g**). Lines indicate ovarian response index in two successive IVF treatments of individual women, with decrease in blue and increase in red.

Groups were compared with chi-square test, Student's t-test and Mann-Whitney test where appropriate. Age-related change in ovarian response (Figure 2) was assessed with non-linear regression analysis using the *nls* function of R. The analysis was based on the first ovarian stimulation cycle, in case of women who had undergone several treatments. The expected ovarian response after unilateral oophorectomy (Table 1) was derived as follows: First, the advancement of reproductive age was predicted for each unilaterally oophorectomised woman, assuming the total ovarian follicle pool model of ovarian ageing and taking into account the age at surgery. Second, the ovarian response for a woman matching this predicted reproductive age was calculated from the regression model established for Figure 2. In order to assess longitudinal changes in ovarian response (Figure 3), the analysis was limited to a subset of women who underwent at least two treatments>12 months apart. Yearly change in ovarian response was calculated for each woman and regression analysis with nls was used to assess relation of yearly response change with age. P<0.05 was considered statistically significant.

Statistical analysis was performed using the R program package version 2.14 and Statistical Package for Social Sciences (SPSS version 18) for Windows (SPSS Inc., Chicago, IL).

## Results

We applied alternative functions of ovarian follicle decline to histological data from Kelsey and Wallace [4] (Figure S1). Faddy's decay curve most faithfully fitted the dataset (Figure 1 and S1 in File S1), showing a progressive decline in follicle reserve with age. We then examined two models of ovarian ageing following unilateral oophorectomy.

The conventional total ovarian follicle pool model of ovarian ageing, assumes a single ovarian follicle pool, comprising of two ovaries with inter-ovarian endocrine signalling. This model predicts that a reduction of the pool by half would advance ovarian age to that of a woman with a naturally diminished ovarian pool of an equivalent size (Figure 1). This would lead to an equivalent rate of decline in ovarian follicle reserve, but from an earlier timepoint. This would ultimately result in a significantly advanced menopause, the magnitude of which would depend on the age at oophorectomy (Figure 1 a–c - green line). By contrast, an alternative model would predict a decline after oophorectomy from a halved follicle pool size, at a rate equivalent to half that of an unoperated woman. As such, a more gradual decline in ovarian reserve results in a modest difference in the expected timing of menopause, and with little effect of age of oophorectomy. (Figure 1 a–c – red line).

We quantified ovarian response to controlled ovarian stimulation cycles, as part of assisted reproduction treatments in women who had undergone unilateral oophorectomy or were not operated (Table S2 in File S1). The total dose of FSH administered in women after unilateral oophorectomy was higher compared to women with two ovaries (Table S2 in File S1). Individual variations in gonadotropin dosage were accounted for by expressing ovarian response as a ratio of the number of collected oocytes per 1000 IU total FSH dose. Removal of an ovary by oophorectomy resulted in a halving of average ovarian response from 5.10 to 2.57 oocytes collected per 1000 IU of FSH administered to youngest age group of women between 21 and 32 years of age (Table 1). Thus, halving ovarian follicle number led to an approximate halving of total ovarian response, consistent with an alternative model of ovarian ageing.

We used data published by Hansen and Knowlton [2], [23] to estimate the expected advancement in reproductive age, as predicted by the total ovarian follicle pool model, for patients that had undergone unilateral oophorectomy. Predicted advancement in reproductive age averaged 5 years overall, and varied according to age at oophorectomy (Table 1), We calculated expected ovarian response using predicted ages from our data of ovarian response in unoperated women (Table 1). We then compared the expected ovarian response in unilaterally oophorectomised women to observed values using a paired-sample t-test, and found that unilaterally oophorectomised women had a significantly lower than expected response in the youngest age group (2.57 vs 3.77), and across all age groups (age-groups (2.90 vs 2.27). This argues against the total ovarian follicle pool model of ovarian ageing and instead suggests that ovarian age follows an alternative model (Table 1).

An alternative model could be explained by two possible mechanisms: a lack of inter-ovarian signalling between the ovaries – giving rise to two independent single ovarian follicle pools, or by an age-dependent extra-ovarian regulation that controls the rate of ovarian follicle pool depletion. An inter-ovarian signal-independent mechanism would predict a halving in ovarian response after unilateral oophorectomy, and a decline in response at half the rate of a woman with two ovaries. However, an age-dependent mechanism could give rise to a range of ovarian response dynamics during ageing.

Our data shows that, in contrast to younger ages, ovarian response exceeds that predicted by the total ovarian follicle pool model in the oldest age group (Table 1). Furthermore, ovarian response is also greater than predicted by an inter-ovarian signal-independent explanation of the alternative ovarian ageing model in patients over 35 (2.17 vs 3.3/2 = 1.65) (Table 1). These data display shifting ovarian response dynamics with age, supporting a role for age-dependent regulation.

In women with both ovaries in place (n = 6747), ovarian response index was best predicted by a two-piece exponential decay model. In this model, ovarian response decreased at a yearly rate of b = −0.03 (SE 0.008; P = 0.002), followed by a yearly decrease of b = −0.08 (SE 0.006; P<0.001) after 32.1 (SE 0.67) years of age (Figure 2a). This relationship is consistent with published declines in ovarian reserve [15], as well as estimators of ovarian reserve, such as antral follicle count and anti-Müllerian hormone (AMH) [24]. However, in women over 32 years of age, who have undergone unilateral oophorectomy (n = 59), there was a non-significant decline in ovarian response in relation to age (b = −0.01; SE 0.05; P = 0.82; Figure 2b) and time since oophorectomy (b = 0.009; SE 0.02; P = 0.60; Figure 2c). This suggests that the dynamics of ovarian response are altered with age, rather than simply a function of remaining follicle number.

To account for individual dynamics of ovarian response and possible age effects, we examined longitudinal changes in ovarian responses between successive assisted reproductive treatments, with a minimum inter-treatment period of 12 months (Figure 3). In women with two ovaries (n = 1604), ovarian response declined at a yearly rate of b = − 0.02 (SE 0.02) up to 32 years of age, after which, there was an increased rate of decline (Figure 3a–c; b = −0.07, SE 0.01; P = 0.03). Women who had undergone a unilateral oophorectomy (n = 20) showed a decline in ovarian response (Figure 3b–f; b = −0.10, SE 0.10) under the age of 32 years However, those above 32 years of age experienced a significant change to a yearly increase in response (b = 0.25, SE 0.10; P = 0.02). The individual change in ovarian response was not significantly related to time since oophorectomy (b = −0.01; P = 0.16). These data show similarities between decline of ovarian response with ageing in women with both ovaries intact (Figure 3b), and decline with time since oophorectomy in women with a halved ovarian follicle pool (Figure 3g). However, the observed increase in response in older operated women (Figure 3e), suggests an age-dependent effect, which results in a preservation of ovarian function in later reproductive years.

**Figure 4:**
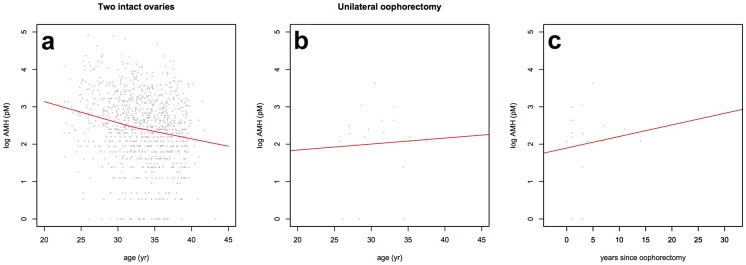
Anti-Müllerian Hormone concentration after unilateral oophorectomy. Serum AMH concentrations in women with two ovaries (**a**) and one ovary (**b**), according to age. AMH in women with one ovary according to time since unilateral oophorectomy. The ovarian dynamics match the setting of Figure 3.

Serum AMH concentrations also support this finding of increased follicle growth in older women who have undergone oophorectomy (Figure 4). Women with intact ovaries showed a decline in AMH levels with age (Figure 4a; n = 1054), whereas those who had undergone oophorectomy showed increased AMH levels at later reproductive age, indicative of larger numbers of small growing follicles (Figure 4b,c; n = 19). Together these data suggest that ovarian ageing is independent of the overall ovarian follicle pool, and that reproductive decline is first regulated by intra-ovarian paracrine signalling, and later by age-dependent regulation of ovarian function.

## Discussion

Females, born with a vast stock of oocytes, face sterility little more than halfway through their modern lifespan, having only ovulated several hundred oocytes [2], [6]. The differing rates at which body tissues age raise questions over the mechanisms and regulation of ageing in reproductive and non-reproductive organs [9]. These questions are no more profound than in relation to the ovaries, where control of follicle and oocyte loss over the course of the human reproductive span is a complex interplay between rates of recruitment, growth, selection, and atresia [6].

The data presented here are in opposition to a total ovarian follicle pool model of reproductive ageing that includes both intra- and inter-ovarian signalling, without any extra-ovarian age-dependent signalling. Ovarian response fell to lower levels than predicted by this model after oophorectomy, rates of decline in ovarian response were lower than expected, and response in older women was increased. These data are therefore consistent with the regulation of reproductive ageing by two possible alternative mechanisms.

The simplest explanation would involve independent intra-ovarian regulation of the two ovaries, in the absence of inter-ovarian signalling. In this scenario, removal of an ovary would leave the remaining organ to continue to function as previously, resulting in a halved ovarian response and a more gradual decline in this response over time, as compared to an unoperated control. An alternative mechanism would rely upon an additional layer of age-dependent regulation, through extra-ovarian signalling, in the presence of both the intra- and inter-ovarian signalling.

Our ovarian response data show that follicle dynamics are altered in later reproductive life, giving rise to maintenance of ovarian function, despite a halving of the overall ovarian follicle pool by unilateral oophorectomy. This suggests a present and increasingly important role for age-dependent extra-ovarian regulation of reproduction in later reproductive life. However, it is conceivable that the maintenance in ovarian response occurs as a result of follicle numbers falling below a threshold, which is reached earlier in unilaterally oophorectomised women. However, ovarian response in unilaterally oophorectomised women over the age of 35 is in excess of half the response of unoperated women of the same age, suggesting that age is more important than remaining follicle number in this older age group.

Our conclusions are supported by biochemical data, collected here, showing an increase or maintenance of serum AMH levels in older oophorectomised women, indicative of growing follicles, and normally associated with ovarian reserve [25]. Given the unlikelihood of *de novo* follicle formation in adult women [26], [27], [28], increasing AMH may be explained by changing follicle dynamics. A shift in the proportion of resting follicles entering the growing cohort, as opposed to undergoing atresia, may result in increased AMH levels and ovarian response maintenance in older women.

Our conclusions are further supported by studies investigating the timing of menopause in oophorectomised women. Removal of a single ovary in adults leads to a modest shortening of time to menopause by 1–2 years [29], [30], [31], as opposed to a predicted advancement of up to ten years that might be associated with a total ovarian follicle pool model of ovarian ageing (Figure 1b).

The age at which the ovarian follicle pool is reduced may also be important to the long-term regulation of reproductive decline. A reduction early in life may have more severe consequences for later ovarian function due to the relatively large number of follicles in a young girl, compared to that of an older woman. Indeed, in studies of childhood cancer survivors, both chemotherapy and oophorectomy advanced menopause by large degrees (four years for alkylating agents and seven years for unilateral oophorectomy) [32], compared to a single year in studies including unilateral oophorectomies of older women [30], [31]. The remaining number of follicles entering a later, increasingly age-dependent stage of ovarian decline may consequently be insufficient to maintain ovarian function, leading to an early loss of ovarian response and menopause.

Little is known about extra-ovarian age-dependent regulation of reproductive ageing, as proposed here. Suppressors of follicle activation are predominantly believed to have autocrine or paracrine functions. Indeed, several lines of evidence suggest that the effect of AMH is restricted locally. For example, AMH only inhibits the development of the Müllerian ducts in the foetus in an ipsilateral fashion [33], and *in vitro* activation of human ovarian follicles was only inhibited by higher than physiological levels of AMH [34]. However, research in lower organisms has identified a number of signalling pathways that differentially regulate organismal and reproductive ageing [35]. *C. elegans* mutants of the well characterised insulin/insulin-like growth factor (IGF-1) pathway display increased longevity and reproductive span [35]. By contrast, TGF-β signalling has been demonstrated to control reproductive ageing, whilst having a limited effect on lifespan [36].

A number of possible pathways signalling between the soma and germline have also been demonstrated [37]. Recently, gonadotropin-releasing hormone (GnRH), usually associated with the regulation of sex hormones, has been shown to affect lifespan in mice. It is therefore conceivable that GnRH, or an associated endocrine factor, may function as a signal that integrates reproductive and organismal ageing in mammals [38]. Furthermore, treatment with GnRH-agonists has been suggested to protect resumption of menstruation and ovulation in premenopausal women exposed to chemotherapy for cancer [39] .

Our data may also have implications for clinical practice. We have shown that ovarian response to FSH during IVF treatment is halved in women who have had undergone surgical removal of a single ovary younger than 32 years. However, in older age the response is unexpectedly maintained, compared to predictions based on the total ovarian follicle pool model. This information is of importance in fertility planning for those undergoing ovarian surgery.

In conclusion, we have found that surgical removal of half of the ovarian reserve leads to a dramatic fall in overall ovarian response, but response per ovary is maintained in young women. In contrast to predictions drawn from the total ovarian follicle pool model of reproductive ageing, reduction of ovarian reserve does not lead to a premature decline in ovarian response. Maintenance of ovarian response in later reproductive years suggests an important and increasing role for extra-ovarian age-dependent regulation of reproductive decline during the normal ageing process. Together, our data suggest that decline in ovarian function is controlled by a combination of intra-ovarian paracrine signalling and extra-ovarian age-dependent factors. This highlights the need for further research to identify fundamental pathways involved in the integration of organismal and reproductive ageing in humans.

## Supporting Information

Figure S1 **Models of ovarian follicle depletion.** At ages 10 years (c,h,m,r), 20 years (d,i,n,s), and 35 years (e,j,o,t), assuming alternative (red) or the total ovarian follicle pool (green) follicle loss. (**a–e**) Faddy's differential equation (equation No. 4) [14]; (**f–j**) Faddy's 'broken stick' regression (i.e. piecewise log-linear regression, Fig 1) [14]; (**k–o**); Hansen's power model [2]; (**p–t**) Wallace-Kelsey model (5-parameter asymmetric double-Gaussian cumulative curve) [4].
(TIF)

File S1 **Supporting tables.** Table S1, Indications for unilateral oophorectomy. Table S2, Clinical characteristics and assisted reproduction treatment in women with unilateral oophorectomy and in control patients.
(DOC)
