# Supplementary figures and images for: Female Reproductive Decline Is Determined by Remaining Ovarian Reserve and Age
Source: PLoS One. 2014 Oct 13;9(10):e108343. doi: 10.1371/journal.pone.0108343 (PMC4195570; doi:10.1371/journal.pone.0108343)

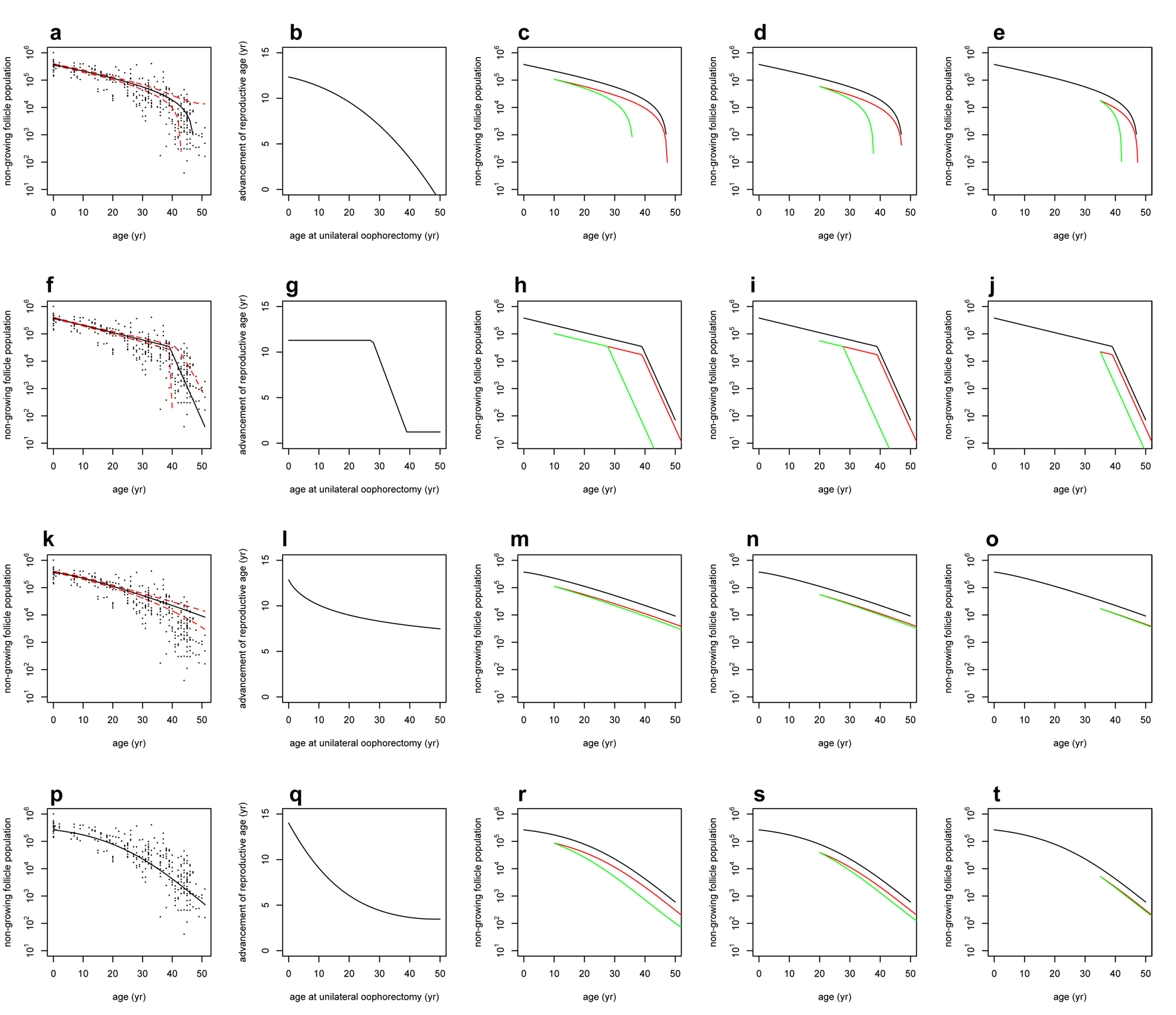

Supplement: Figure S1 — Models of ovarian follicle depletion. At ages 10 years (c,h,m,r), 20 years (d,i,n,s), and 35 years (e,j,o,t), assuming alternative (red) or the total ovarian follicle pool (green) follicle loss. (a–e) Faddy's differential equation (equation No. 4) [14]; (f–j) Faddy's 'broken stick' regression (i.e. piecewise log-linear regression, Fig 1) [14]; (k–o); Hansen's power model [2]; (p–t) Wallace-Kelsey model (5-parameter asymmetric double-Gaussian cumulative curve) [4]. (TIF) [file pone.0108343.s001.tif]
