# Supplementary material for: Female Reproductive Decline Is Determined by Remaining Ovarian Reserve and Age
Source: PLoS One. 2014 Oct 13;9(10):e108343. doi: 10.1371/journal.pone.0108343 (PMC4195570; doi:10.1371/journal.pone.0108343)
Supplement: File S1 — Supporting tables. Table S1, Indications for unilateral oophorectomy. Table S2, Clinical characteristics and assisted reproduction treatment in women with unilateral oophorectomy and in control patients. (DOC) [file pone.0108343.s002.doc]

**Supplementary materials and methods**

The distributions of age, BMI, or primary infertility were comparable between the groups. Patients who had undergone unilateral oophorectomy had experienced a shorter history of infertility and were more likely to suffer from tubal infertility than patients with both ovaries intact. Conversely, male factor and unexplained infertility were more frequent in the control group, as was treatment with ICSI. The total FSH dose administered was higher in the unilateral oophorectomy group than in the control group, while the number of oocytes retrieved, fertilised oocytes, number of embryos transferred and embryos available for cryopreservation were reduced. There was no difference in fertilization rate and pregnancy rate per oocyte retrieval or embryo transfer.

**Table S1**

| **Diagnosis** | **No.** |
| --- | --- |
| Persistent functional cysts and unspecified cysts | 23 |
| Ovarian endometriosis | 21 |
| Benign tumors (dermoid, teratoma, fibroma, adenoma) | 19 |
| Inflammation (salpingo-oophoritis, abscess, pelvic inflammatory disease) | 8 |
| Ectopic pregnancy | 7 |
| Borderline, atypical, or malignant tumor | 7 |
| Unspecified indication | 12 |

**Table S2**

|  | **Unilateral oophorectomy** | **Control** | **P-value** |
| --- | --- | --- | --- |
| No. of patients | 97 | 6747 |  |
| Age at unilateral oophorectomy (years) | 27.1 (14 – 40, SD 6.5) |  |  |
| Age (years) | 33.0 (25 – 43, SD 3.7) | 33.2 (21 – 46, SD 3.8) | 0.67 |
| BMI (kg/m²) | 23.8 (SD 3.6) | 23.6 (SD 3.9) | 0.58 |
| Duration of infertility (years) | 3.0 (2.0-5.0) | 4.0 (3.0-5.0) | 0.04 |
| Diagnosis (No.)**¹** |  |  |  |
| - Tubal | 34 (35.0%) | 1569 (23.2%) | <0.01 |
| - Male | 26 (26.8%) | 2673 (39.5%) | 0.01 |
| - Endometriosis | 26 (26.8%) | 1470 (21.7%) | 0.2 |
| - Unexplained | 12 (12.4%) | 1570 (23.2%) | 0.01 |
| - Other specific causes | 10 (10.3%) | 354 (5.2%) | 0.03 |
| Primary infertility | 71 (73.2%) | 5378 (79.5%) | 0.12 |
| No. of cycles | 201 | 13800 |  |
| Type of treatment (No.) |  |  |  |
| - IVF | 78.4% | 64.9% | <0.01 |
| - ICSI | 21.6% | 35.1% | <0.01 |
| Total FSH dose (IU) | 2250 (1650 – 3037.50) | 1800 (1500 – 2450) | <0.01 |
| Mean no. of collected oocytes | 6.1 (SD 4.6) | 9.0 (SD 5.7) | <0.01 |
| Diploid fertilized zygotes | 3.64 (SD 3.7) | 4.9 (SD 3.8) | <0.01 |
| Diploid fertilization rate | 3.6/6.1 (59%) | 4.9/9.0 (54.4%) |  |
| No. of transferred embryos | 1.2 (SD 0.8) | 1.4 (SD 0.7) | 0.01 |
| No. of cryopreserved embryos | 0.51 (SD 1.3) | 0.90 (2.0) | 0.055 |
| Pregnancy rate per ovum pick up | 26.8% | 33.6% | 0.154 |
| Pregnancy rate per embryo transfer | 34.2% | 38.0% | 0.5 |

**¹** Sum of diagnoses exceeds 100%, because of co-morbidities in some patients.
